# Case of Poisoning by Opium

**Published:** 1845-01

**Authors:** 


### Case of Poisoning by Opium. By *Alfred S. Taylor.* (Guy's Hospital Reports, October, 1844.)

This is one of the numerous instances of death being caused through the administration of opium to children by nurses. The form employed was unusual, namely, an infusion, made by pouring warm water into a bottle containing opium, and shaking the mixture well up. The child (æt. 14

months) had had slight diarrhœa, and, being restless, more than three teaspoonfuls of this fluid were administered to it in a brief space of time. It soon became drowsy, and continued comatose, with the exception of a short interval, until its death, which occurred eighteen hours after the administration of the drug. The chief post-mortem appearance consisted in a very congested condition of the blood-vessels of the brain, unaccompanied by any effusion. A portion of the mixture employed, being tested with nitric acid and sesquichloride of iron, gave unequivocal signs of the presence of opium. A small portion of the contents of the stomach were analysed, but no opium detected therein; a fact nowise surprising, when the length of time between its being taken and the occurrence of death is considered.

Mr. Taylor observes, that toxicological writers have not alluded to the degree of *solubility* of opium in water, although this is a question likely to be put to a witness occasionally. By experiment he found that boiling water poured upon powdered opium, had, after twenty hours, taken up 4 per cent. of its weight, and from 2 to 4 per cent. when the common extract sliced was employed. The dregs still retained poisonous properties, capable of extraction by new infusion or decoction.

*Tests.*—Where opium cannot be detected by the smell the author prefers the sesqui-chloride of iron as a trial test, discovering as it does meconic acid in $\frac{1}{160}$ of a gr. of opium.

"It might be supposed, that if, on adding strong nitric acid to a portion of the liquid, a bright red colour resulted, this would be a sufficient indication of the presence of morphia, and therefore of opium: but a serious mistake might be committed in such a case, unless the operator had previously employed the iron test, and determined the presence of meconic acid in the liquid. It is worthy of remark that the nitric acid test, while it destroys the colour given by the meconate of iron (a dark red), will bring out, when added to excess to the same portion of liquid, the peculiar bright amber-red tint which it is known to give in a solution of morphia. The tests for meconic acid and morphia may thus be applied to one quantity of liquid."

Mr. Taylor, after explaining the fallacies these tests, and that of iodic acid, may give rise to, next describes some experiments he instituted for the discovery of the smallest quantity of *morphia* which can be detected by each. He found that nitric acid detected $\frac{1}{15}$ gr. of muriate of morphia, diluted in 300 parts by weight of water; sesqui-chlor. iron detected the $\frac{1}{11}$ gr. in 231 parts of water; and iodic acid the $\frac{1}{100}$ gr. in 1300 parts of water. Thus *iodic acid* is by far the most delicate test, discovering as it does morphia in less than $\frac{1}{5}$ of a grain of opium; but it is also the one most open to fallacy, and cannot be employed in coloured organic liquids, containing these small quantities. Practically its utility is far less than would be anticipated from the result of experiments upon the pure salts of morphia. Other experiments were performed for the purpose of ascertaining how small a quantity of *meconic acid* need be present in a fluid, to admit of its separation by acetate of lead, and subsequent identification by sesqui-chl. of iron. No precipitate of meconate of lead occurred when the proportion of meconic acid was less than $\frac{1}{48}$ gr. *i. e.* about 0·34 gr. common opium. Unless, indeed, soluble matter of several grains of opium exists in the liquid for analysis, it will be difficult to obtain meconic acid and morphia separately. The *iron test* for *meconic acid* is far more delicate than any of the tests for morphia, and is much less liable to be interfered with. The $\frac{1}{10}$ gr. or smallest visible portion of solid meconic acid is easily detected when free, while in solution, in a small quantity of liquid, even $\frac{1}{500}$ gr. may be discovered. Thus, the presence of this acid may be determined in a liquid from a much smaller quantity than would suffice to form a separable precipitate of meconate of lead; for, while for this latter $\frac{1}{3}$ of a grain of opium is required, less than $\frac{1}{100}$ grain suffices for the exhibition of the acid by the direct application of the iron test. The procural of the precipitate of meconate of lead does not increase the certainty of the iron test, but merely enables us to obtain the meconic acid in a concentrated and solid form.

